# Effect of topical berberine in murine cutaneous leishmaniasis lesions

**DOI:** 10.1093/jac/dkac007

**Published:** 2022-01-28

**Authors:** Alba Calvo, Esther Moreno, Irati Aldalur, Carmen Sanmartín, Esther Larrea, Elena González-Peñas, Juan Manuel Irache, Socorro Espuelas

**Affiliations:** 1 ISTUN Institute of Tropical Health, University of Navarra, Irunlarrea 1, 31008, Pamplona, Spain; 2 Chemistry and Pharmaceutical Technology Department, University of Navarra, Irunlarrea 1, 31008, Pamplona, Spain; 3 Navarra Institute for Health Research, IdisNA, Pamplona, Spain

## Abstract

**Objectives:**

More effective topical treatments remain an unmet need for the localized forms of cutaneous leishmaniasis (CL). The aim of this study was to evaluate the efficacy and safety of a topical berberine cream in BALB/c mice infected with *Leishmania major* parasites.

**Methods:**

A cream containing 0.5% berberine-β-glycerophosphate salt and 2.5% menthol was prepared. Its physicochemical and stability properties were determined. The cream was evaluated for its capacity to reduce lesion size and parasitic load as well as to promote wound healing after twice-a-day administration for 35 days. Clinical biochemical profile was used for estimating off-target effects. *In vitro* time-to-kill curves in *L. major*-infected macrophages and skin and plasma pharmacokinetics were determined, aiming to establish pharmacokinetic/pharmacodynamic relationships.

**Results:**

The cream was stable at 40°C for 3 months and at 4°C for at least 8 months. It was able to halt lesion progression in all treated mice. At the end of treatment, parasite load in the skin was reduced by 99.9% (4 log) and genes involved in the wound healing process were up-regulated compared with untreated mice.

The observed effects were higher than expected from *in vitro* time-to-kill kinetic and plasma berberine concentrations, which ranged between 0.07 and 0.22 μM.

**Conclusions:**

The twice-a-day administration of a topical berberine cream was safe, able to stop parasite progression and improved the appearance of skin CL lesions. The relationship between drug plasma levels and *in vivo* effect was unclear.

## Introduction

Leishmaniasis is considered a major public health problem because 1 billion individuals are at risk of infection worldwide. Among the different forms of leishmaniasis, cutaneous leishmaniasis (CL) is the most common type and its prevalence has almost doubled from 2.1 million cases in 2002 to nearly 4 million cases in 2015.^1^ For decades, pentavalent antimony compounds, administered IV or intralesionally, or topical paromomycin have been the first-line treatment for CL.^2^ However, variation in the clinical response has been a persistent problem over the past 50 years.^3^ With these issues, and according to the WHO, the search for better topical treatments of CL is a priority for their multiple advantages.^4^

Natural products have played a significant role in the drug discovery process throughout the last century. Among them, berberine chloride [1,8,13α-tetra-hydro-9,10-demethoxy-2,3-(methyl-ene-dioxy)-berberium chloride], an isoquinoline alkaloid, has demonstrated pharmacological activity against several diseases, including leishmaniasis.^5,6^ Moreover, it is currently being tested in Phase IV clinical trials for hyperglycaemia and metabolic syndrome and it is approved as an over-the-counter (OTC) drug to treat gastrointestinal infections in China.^7^ This status may greatly accelerate its use for other therapeutic applications (therapeutic switching), especially for topical use.

The purpose of this work was assessment of the efficacy of berberine by the topical route in a murine model of CL, encouraged by the following attributes: (i) berberine has shown high intrinsic antileishmanial activity (EC_50_ of 1 μM against *Leishmania donovani* intracellular amastigotes) and excellent selectivity index (SI) of >125 *in vitro*;^8,9^ (ii) berberine has anti-inflammatory activity^6^ and immunomodulatory properties, mainly mediated by the mitogen-activated protein kinase (MAPK) pathway.^8^ Thus, its antileishmanial and anti-inflammatory dual effect could favour the healing of CL lesions without scarring; (iii) the low molecular weight (around 300 Da) and slightly hydrophilic logP of berberine^10^ should not impede its permeation through damaged skin in CL lesions,^11^ in which the stratum corneum is partially removed;^12^ (iv) berberine suffers rapid hepatic clearance^13^ that can avoid systemic accumulation and side effects upon absorption from skin; and (v) berberine has a low cost. The terpene menthol was incorporated in the topical formulation at 2.5%, a concentration that, despite needing precaution,^14^ can be used. Furthermore, the antileishmanial activity of this terpenoid^15^ could join that of berberine. In addition to this effect, the role of menthol as a permeation enhancer of berberine has been previously reported.^16^ Although infection increased the skin permeability,^11^ such a type of compound could still be needed in a formulation,^17^ as infected macrophages are deeply located in the dermis, surrounded by granulomatous tissue.

Overall, we evaluated berberine efficacy in *Leishmania major*-infected BALB/c mice in terms of both parasite clearance and inflammatory response modulation. Skin and plasma berberine pharmacokinetics (PK) were also determined to establish PK/pharmacodynamic (PK/PD) relationships.

## Materials and methods

### In vitro time-to-kill curves, cytotoxicity and berberine-β -glycerophosphate/menthol combination studies

Toxicity of berberine-β-glycerophosphate and menthol was assessed in bone marrow-derived macrophages (BMDMs) grown in DMEM by the MTT assay, after 48 h. The activity of berberine-β-glycerophosphate and menthol, either alone or in combination, was then evaluated against intracellular *L. major* amastigotes at different incubation times (24, 48 and 72 h) by the back transformation assay (BTA), as previously reported,^18^ using a ratio of 10:1 (parasites:macrophages) and allowing parasite infection overnight. The effective or cytotoxic concentration values (EC_50_, EC_90_ and CC_50_) were obtained by fitting the data to a dose–inhibition sigmoid curve using GraphPad Prism 7.0 software (GraphPad Software Inc., San Diego, CA, USA). SI was calculated as the ratio between cytotoxicity (CC_50_) against BMDM and activity (EC_50_) against *Leishmania* amastigotes. In order to evaluate whether menthol had no interaction, synergy or antagonism in combination with berberine-β-glycerophosphate, an FIC index (FICI) was calculated after 48 h of treatment.^19^ See the Supplementary methods for more details (*BTA* and *Drug combination studies* sections), available as Supplementary data at *JAC* Online.

Berberine protein binding in the *in vitro* assay medium (DMEM containing 10% heat-inactivated FBS) was estimated by the ultracentrifugation technique, as previously described^20^ and detailed in the Supplementary methods (*In vitro determination of berberine binding to plasma and culture medium proteins* section). This percentage was used to determine the *f*EC_50_ and *f*EC_90_ (*in vitro* EC_50_ and EC_90_ against *Leishmania* corrected for protein binding), on the basis of the obtained EC_50_ and EC_90_ values.

### Preparation, characterization and stability of berberine-β-glycerophosphate cream

To obtain berberine-β-glycerophosphate cream, 12 g of the oily phase containing cetyl alcohol (1.5%), stearic acid (2.5%), solid paraffin (5%), liquid paraffin (7.5%), glyceryl monostearate (11%) and menthol (2.5%) was melted in a mortar at 70°C. Water (enough for 40 g), berberine-β-glycerophosphate salt (0.5%), Polysorbate^®^ 20 (2.5%) and preservatives [Nipagin^TM^ (0.16%) and EDTA (0.07%)] were then heated at 70°C and poured onto the oily phase under agitation until the mixture was cooled. Cream without berberine-β-glycerophosphate was also prepared, substituting the amount of salt by water, and identified as vehicle. Their physicochemical properties were evaluated and stability studies were carried out. More details are provided in the Supplementary methods (*Characterization and stability of berberine-β-glycerophosphate cream* section).

### Animals

This study was conducted in BALB/c mice (Harlan, Spain) housed in groups of five in plastic cages under controlled environmental conditions (12:12 h light/dark cycle and 22 ± 2°C), according to ethical standards approved by the Animal Ethics Committee of the University of Navarra, in strict accordance with the European legislation in animal experiments (protocol number 100-19).

### Ex vivo permeation and penetration studies in healthy mouse skin

Permeation studies under infinite conditions were carried out in freshly excised mouse skin according to the Organisation for Economic Cooperation and Development (OECD) guideline 428^21^ during 24 h using Franz diffusion cells. The receptor compartments were filled with a PBS solution, enough to ensure sink conditions. Full-thickness excised female mouse skin pieces were placed between the donor and receptor compartments. Receptor fluid samples were taken at determined times (0.5, 1, 2, 4, 6, 8, 10 and 24 h). Berberine in the receptor compartment and in the skin was quantified using HPLC-MS/MS. The flux of drug permeated (*Jss*, μg/cm^2^·h), lag time (h) and permeability coefficient (*Kp*, cm/h) were then calculated. More details are provided in the Supplementary methods (*Ex vivo permeation studies in healthy mouse skin* section).

### In vivo efficacy of berberine-β-glycerophosphate cream in L. major-infected BALB/c mice

After 2 weeks of infection (lesion size around 6 mm^2^), mice (*n* = 5–6) were left untreated or topically treated with berberine-β-glycerophosphate and its vehicle. Mice received 7.5 mg/kg of berberine-β-glycerophosphate twice a day (15 mg/kg total dose) during 35 consecutive days. Lesions were measured every 4 days with a digital calliper. Three days after the end of treatment, parasite load in popliteal lymph nodes (LNs) and skin lesions was quantified after DNA extraction by quantitative RT–PCR (qRT–PCR). Cytokine expression in skin lesions was also assessed at the end of treatment by qRT–PCR, after RNA extraction. More details are provided in the Supplementary methods (*DNA extraction and parasite quantification* and *RNA extraction and cytokine expression* sections and Table S1).

### PK and dermatokinetic (DK) studies during treatment

Blood from non-infected and *L. major*-infected mice (*n* = 6) was collected from the submandibular plexus of mice at determined timepoints after the first daily application on Days 1 and 10. At the same timepoints on Day 1, lesions were carefully washed with methanol (to remove the remaining formulation) and berberine accumulation in the skin lesions was analysed by HPLC-MS/MS. Also, blood was collected on Days 3, 7, 13, 18, 22, 30 and 35. For estimation of PK parameters, healthy mice were injected IV with 7.5 mg/kg berberine (previously dissolved in type I water with 5% glucose at 1 mg/mL) and blood samples were collected at 0.25, 0.5, 1, 4 and 24 h. Plasma concentrations were obtained by centrifugation of blood at 6000 g for 10 min and berberine was quantified by HPLC-MS/MS. PK parameters were calculated using non-compartmental analysis (NCA) and the Excel PKSolver program.^22^ More details are provided in the Supplementary methods (*Berberine extraction from plasma and skin samples* and *Quantification of berberine by HPLC-MS/MS* sections).

Berberine binding to plasma proteins was determined after incubating different concentrations of berberine diluted in mouse plasma (from 50 to 1000 ng/mL), using the same procedure described for determining the drug binding in cell culture medium and detailed in the Supplementary methods (*In vitro determination of berberine binding to plasma and culture medium proteins* section).

### Histological analysis and immunohistochemistry

At the end of treatments, skin fragments were formalin-fixed, paraffin-embedded, cut in 3 μm thick sections and stained with haematoxylin and eosin (HE). Immunohistochemistry for NIMP-R14 (neutrophils), F4/80 (macrophages) and CD3 (lymphocytes) was also carried out. Digital images were scanned using a digital microscope system (Aperio ScanScope CS2, Leica Biosystems). For immunohistochemistry analysis, the percentage of area stained in each image was then quantified by Fiji 2.0. software. More details are provided in the Supplementary methods (*Immunohistochemistry studies* section).

### Biochemical analysis after topical berberine-β-glycerophosphate cream administration: in vivo toxicity studies

Biochemical analysis was performed in non-infected and *L. major*-infected mice. At the end of treatment, blood samples were collected from the submandibular vein in order to evaluate renal and liver toxicity. ALT, AST, creatinine (CRE), alkaline phosphatase (ALP), glucose, total cholesterol (CHO), HDL cholesterol (HDL), LDL cholesterol (LDL), urea (BUN) and triglyceride (TRIG) concentrations were measured in serum in a Cobas^®^ biochemistry analyser (Roche). Non-treated mice were bled for comparison.

### Statistical analysis

Statistical analyses between three groups were performed by Kruskal–Wallis (non-parametric) followed by Dunn’s multiple comparisons tests. Differences between two groups were analysed by a non-parametric Mann–Whitney test or by parametric *t*-test, depending on their Shapiro–Wilk normality test. GraphPad Prism7 was used to perform the analysis. Significance was established for *P* values of <0.05.

## Results

### In vitro time-to-kill curves, cytotoxicity and berberine-β-glycerophosphate/menthol combination studies

As presented in Table 1, berberine-β-glycerophosphate showed high activity against *L. major* amastigotes after 48 h of treatment (EC_50_ and EC_90_ of 0.07 and 0.22 μM, respectively) and low cytotoxicity (CC_50_ of 125.3 μM), as an SI of 1790 was determined. Moreover, as several terpenoids have demonstrated antileishmanial activity,^15^ the activity and toxicity of menthol either alone or in combination with berberine-β-glycerophosphate was calculated to determine whether some interaction between the two compounds occurred. After 48 h incubation time, menthol showed low antileishmanial activity (EC_50_ and EC_90_ of 80.8 and 783.3 μM, respectively) and an SI value of 6.9 (Table 1). Although an FICI value of 0.95 was found between berberine-β-glycerophosphate and menthol, which indicated no interaction between the two compounds (Table 1), lower EC_50_ values were calculated in the dose–response curves of berberine-β-glycerophosphate when menthol (10 or 50 μM) was added (Figure S1).

**Table 1.** *In vitro* activity against *L. major* amastigotes, cytotoxicity in BMDM and SI of berberine-β-glycerophosphate (BER-GP) and menthol (MNT) after 48 h of treatment

Drug	*L. major* amastigotes		BMDM	SI	FICI
	EC_50_	EC_90_	CC_50_		
BER-GP	0.07 (0.06–0.10)	0.22 (0.16–0.30)	125.3 (107.1–143.4)	1790	0.95
MNT	80.8 (52.5–124.3)	783.3 (429.5–1128.6)	561.3 (338.5–784.1)	6.9	No interaction

EC_50_, EC_90_ and CC50 data are expressed in μM (95% CI), *n* = 3. FICI value was obtained for amastigotes after 48 h of BER-GP treatment combined with MNT (*n* = 3).

**Figure 1.**
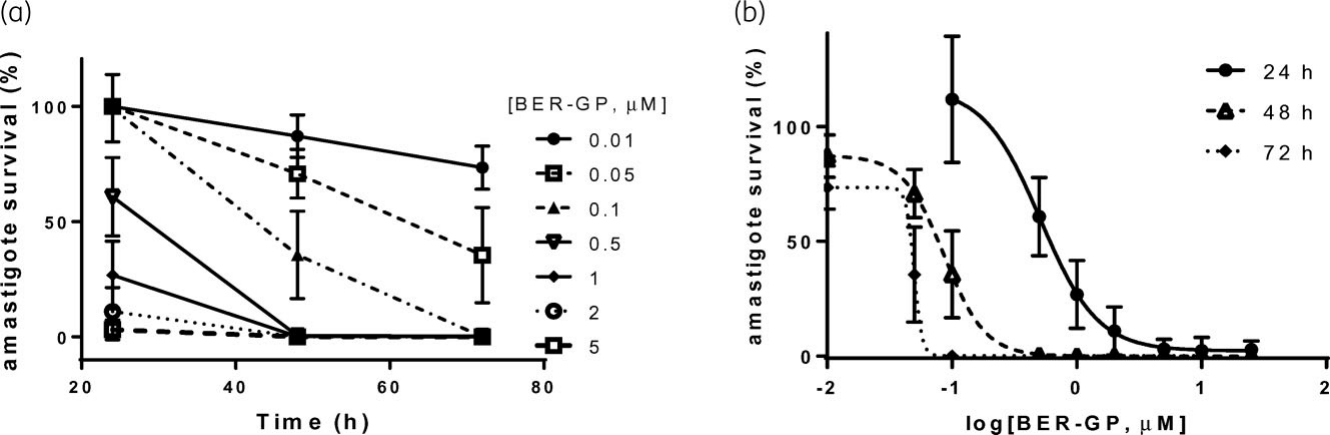
(a) Time-to-kill curves for berberine-β-glycerophosphate (BER-GP) at indicated drug concentrations (μM) and (b) sigmoidal fitting of BER-GP salt dose–response at the indicated incubation times. The effect was determined against *L. major* amastigote-infected macrophages and evaluated by the BTA assay. Data (mean ± SD, *n* = 4) are expressed as amastigote survival (%), calculated on the basis of untreated infected macrophages.

Furthermore, as seen in Figure 1 and Table S2, berberine-β-glycerophosphate reduced amastigote viability in a dose- and time-dependent manner, with lower EC_50_ and EC_90_ values at longer times of treatment. In fact, EC_50_ and EC_90_ values of 0.02 and 0.12 μM, respectively, were obtained for berberine-β-glycerophosphate after 72 h of treatment (Table S2), compared with values of 0.56 and 1.52 μM after 24 h. Moreover, 0.01 and 0.05 μM concentrations of berberine-β-glycerophosphate were unable to achieve maximal amastigote reduction (Figure 1a) after 72 h. Concentrations of 0.1, 0.5 and 1 μM produced complete removal of parasites at long incubation times (72 h). In contrast, 2 μM or higher concentrations of berberine-β-glycerophosphate exhibited maximal activity after 24 h. Overall, it seems that a berberine concentration of 2 μM or higher would be required at the target site (dermal macrophages) to achieve maximal and fast effect.

Finally, the obtained values for *in vitro* berberine-β-glycerophosphate activity were corrected according to the extent of drug–protein binding measured in the culture medium (30.7% ± 8.9%), as the activity of a drug is generally accepted to be produced by the unbound drug concentration at the site of action.^23^ These values were 0.01 and 0.08 μM for *f*EC_50_ and *f*EC_90_, respectively, after 72 h (Table S2).

### Characterization of berberine-β-glycerophosphate cream

Creams without (vehicle) or containing berberine-β-glycerophosphate were characterized in terms of viscosity, spreadability and pH (Table S3). Berberine-β-glycerophosphate cream showed higher viscosity (0.97 versus 1.29 Pa·s), lower spreadability (2.5-fold) and lower pH (6.09 versus 4.81) compared with vehicle. Berberine-β-glycerophosphate cream was stable for 3 months at the three different storage temperatures (4°C, 25°C and 40°C) (Table 2). However, after 8 months at 25°C and 40°C, the cream showed signs of exudation at 40°C and the recovery of the drug decreased (79.2% and 73.2%, respectively).

**Table 2.** Stability studies for berberine-β-glycerophosphate (BER-GP) cream over a period of 8 months at different storage temperatures

BER-GP cream	3 months			8 months		
	4°C	25°C	40°C	4°C	25°C	40°C
pH	4.70 ± 0.35	3.79 ± 0.30	4.13 ± 0.16	3.71 ± 0.12	3.67 ± 0.20	3.86 ± 0.25
Spreadability (cm)	0.60 ± 0.07	0.50 ± 0.04	0.70 ± 0.14	0.65 ± 0.15	0.70 ± 0.14	0.85 ± 0.08
BER recovery (%)	99.5 ± 2.1	98.2 ± 1.8	98.2 ± 1.8	100.5 ± 2.2	79.2 ± 1.8	73.2 ± 6.2
Colour	Intense yellow	Intense yellow	Intense yellow	Intense yellow	Pale yellow	Pale yellow
Other organoleptic properties	Smooth, soft, characteristic MNT odour			Smooth, soft, characteristic MNT odour		
Phase separation	No evidence	No evidence	No evidence	No evidence	Yes	Yes
Drug precipitation	No evidence	No evidence	No evidence	No evidence	No evidence	No evidence
Gravitational stability	Yes	Yes	Yes	Yes	Yes	Yes

Data expressed as mean ± SD (*n* = 3).

### Ex vivo permeation studies

*Jss* (from 72.3 to 98.1 ng/cm^2^/h) and *Kp* (from 1.4 × 10^−5^ to 2.0 × 10^−5^ cm/h) of berberine was slightly increased by adding menthol to the cream (Table 3). Although the role of menthol as a permeation enhancer has been previously described,^24^ specifically for this drug in rat skin,^16^ the observed effect was modest (Table 3), probably because of the lower menthol concentration used, compared with the study of Patel *et al.*^16^ (2.5% versus 12.5%). Moreover, berberine in skin was similar in both groups. The creams had long lag times (3 and 2.5 h), as previously observed with other berberine topical formulations.^25^

**Table 3.** *Ex vivo* permeation values obtained for berberine-β-glycerophosphate (BER-GP) creams without or with menthol (MNT) across healthy mouse skin after 24 h

Composition	*Jss* (ng/cm^2^/h)	*Kp* (cm/h)	Lag time (h)	Permeated BER (ng/cm^2^)	BER in skin (ng/mg)
BER-GP (0.5%)	72.3 ± 3.6	1.4 × 10^−5^	3	1533.2 ± 789.8	76.7 ± 19.6
BER-GP (0.5%) + MNT (2.5%)	98.1 ± 13.4	2.0 × 10^−5^	2.5	2366.4 ± 865.1	72.0 ± 17.1

Results are expressed as mean ± SD (*n* = 6). Comparisons between two groups were made by a parametric *t*-test (no significant results).

### In vivo efficacy of topical berberine-β-glycerophosphate cream in L. major-infected BALB/c mice

**Figure 2.**
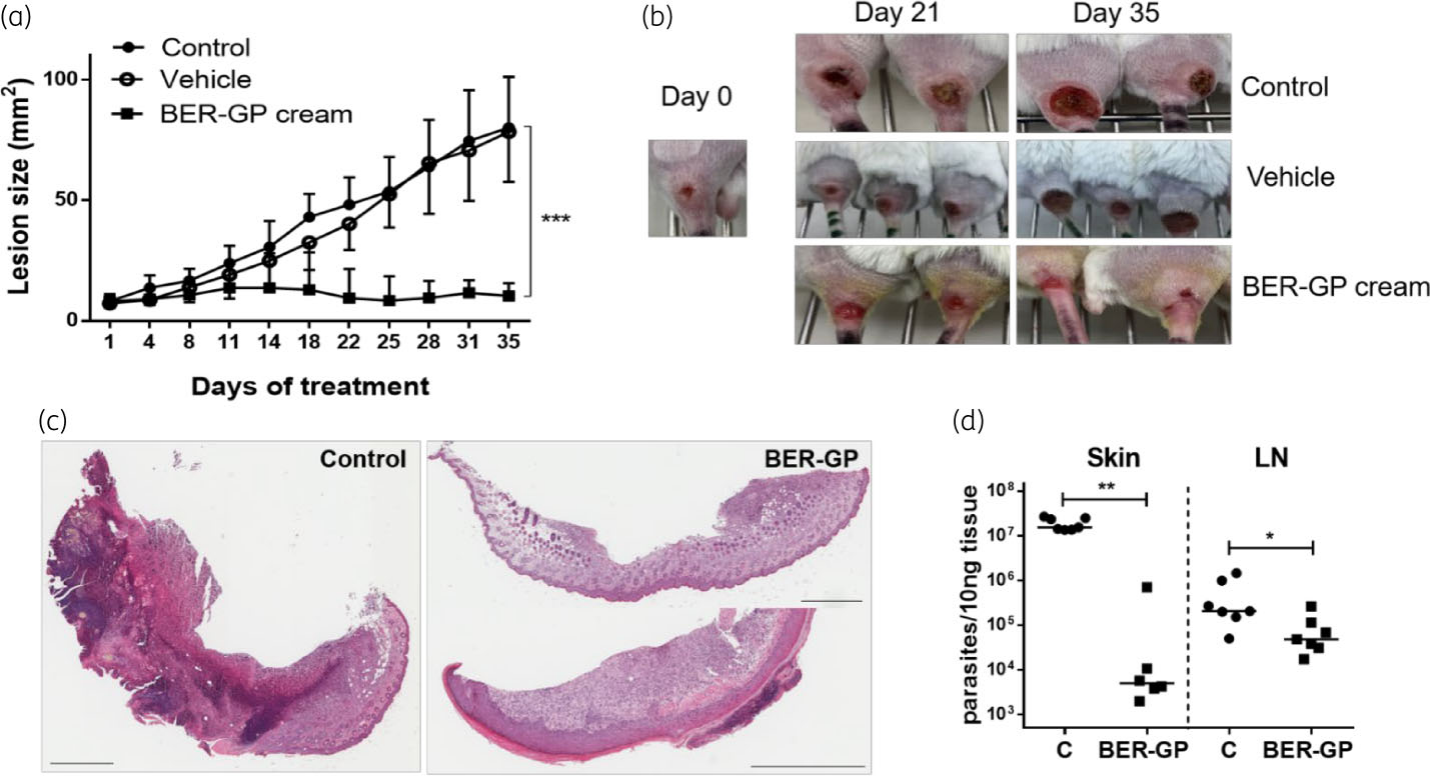
(a and b) Lesion size progression during topical berberine-β-glycerophosphate (BER-GP) cream treatment (control, filled circles; vehicle, open circles; and BER-GP cream, filled squares). (c) Representative images of HE-stained skin sections obtained from a control mouse (infected and non-treated, left) and BER-GP cream treated mice (right). Scale bars: 800 μm. (d) Parasite burden in skin lesions and LNs of *L. major-*infected BALB/c mice after 35 days of topical treatment with BER-GP cream (15 mg/kg daily in two doses, filled squares), compared with non-treated mice (C = control, filled circles). Results are expressed as median (*n* = 6–11 per group). Data were analysed by a non-parametric Mann–Whitney test. * *P <* 0.05, ** *P <* 0.01, *** *P <* 0.001. This figure appears in colour in the online version of *JAC* and in black and white in the print version of *JAC*.

Two weeks after infection, treatments started. The ability of the treatment to halt the lesion growth started to be observed after 3 days of treatment, although significant differences with untreated control lesions only occurred after 18 days. Lesion progression stopped in berberine-β-glycerophosphate-treated mice and lesion sizes were significantly smaller than lesions of non-treated mice (7.8-fold; *P <* 0.001; Figure 2a and b). Moreover, as seen in Figure 2b, lesions treated with berberine-β-glycerophosphate cream were more superficial and hair started to grow. To determine whether menthol presented some leishmanicidal effect *per se*, mice were also treated with vehicle. No reduction in lesion progression was observed (Figure 2a).

Histological analysis of control (infected, untreated; Figure 2c, left) and berberine-β-glycerophosphate cream-treated skin (Figure 2c, right) confirmed the healing of the lesions, although skin was never completely regenerated and foci of necrosis and inflammation persisted. Accordingly, berberine-β-glycerophosphate cream significantly decreased (*P <* 0.01; 99.9%; 4 log) the number of parasites in skin lesions (Figure 2d). Parasite burden in LNs was also significantly reduced (*P <* 0.05; 76.6%; 1 log) after topical application of berberine.

### Immunohistochemistry and gene expression of CL lesions

**Figure 3.**
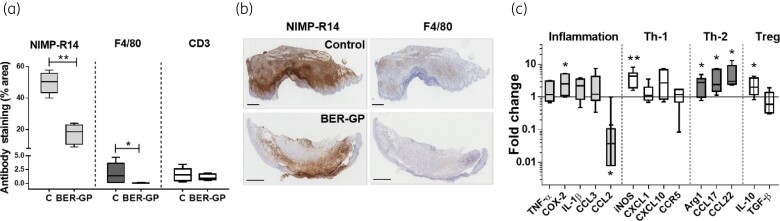
(a) Immunohistochemical analysis in skin lesions of non-treated *L. major-*infected BALB/c mice (C = control), or after 35 days of topical treatment with berberine-β-glycerophosphate (BER-GP) cream for neutrophils (NIMP-14, light grey), macrophages (F4/80, dark grey) and lymphocytes (CD3, white). Boxes represent median (central line) ± 95% CI (upper and lower edges) and whiskers represent minimum and maximum values (*n* = 5 per group). (b) Representative images of skin sections for control (infected untreated mice, top) and mice treated with BER-GP cream (bottom) stained with antibodies against NIMP-R14 (neutrophils) and F4/80 (macrophages). Scale bars: 600 μm. (c) Cytokine expression in skin lesions from *L. major*-infected BALB/c mice after 35 days of topical treatment with BER-GP cream, compared with non-treated mice (C = control), expressed as fold change. Boxes represent median (central line) ± 95% CI (upper and lower edges) and whiskers represent minimum and maximum values (*n* = 5 per group). Data were analysed by a non-parametric Mann–Whitney test. * *P <* 0.05, ** *P <* 0.01. This figure appears in colour in the online version of *JAC* and in black and white in the print version of *JAC*.

Immunohistochemical staining (Figure 3a and b) of skin lesions at the end of berberine-β-glycerophosphate treatment confirmed a significantly lower number of neutrophils (NIMP-R14; *P <* 0.01) and a significant reduction in macrophage (F4/80) infiltration (*P <* 0.05), consistent with the smaller lesion sizes observed after topical berberine-β-glycerophosphate therapy.

On the other hand, berberine-β-glycerophosphate treatment significantly up-regulated the mRNA expression of (iNOS) (*P <* 0.01) as well as COX-2, Arg1, CCL17, CCL22 and IL-10 (*P <* 0.05) (Figure 3c). In contrast, CCL2 appeared down-regulated after treatment (*P <* 0.05). The higher expression of iNOS indicates that treatment with berberine could activate macrophages to produce nitric oxide (NO), leading to the killing of *Leishmania* parasites. Moreover, the up-regulation of Arg1, CCL17, CCL22 and IL-10 and the down-regulation of CCL2 could be in accordance with wound-healing repair mechanisms.

### PK and DK studies

**Figure 4.**
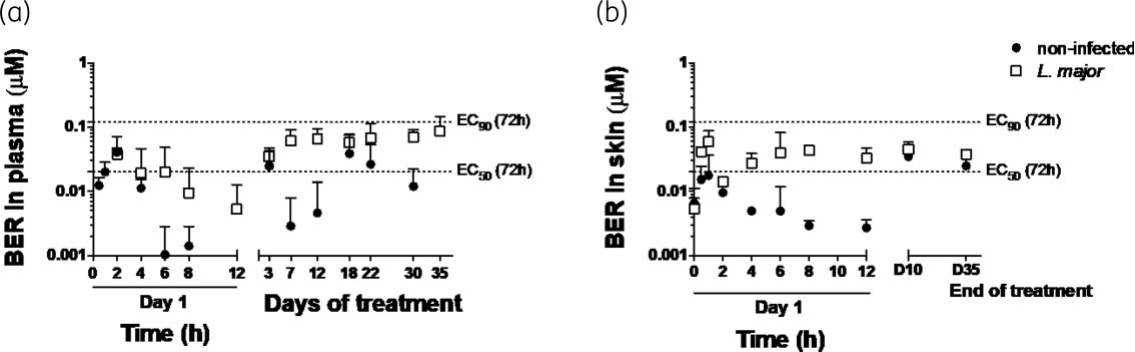
(a) Berberine (BER) plasma concentration at different times (0–8 h) on Day 1 and over the course of the BER-β-glycerophosphate (BER-GP) cream topical treatment in non-infected (filled circles) and *L. major*-infected mice (open squares), measured in blood extracted 12 h after the last daily administration (*C*_trough_). (b) Skin BER levels at different times (0–12 h) on Day 1, Day 10 (D10) and Day 35 (D35), 12 h after the last treatment. Results are expressed as mean ± SD (*n* = 2–4).

Berberine plasma concentration (*C*_trough_) and drug concentration in the total skin homogenates after a single (at Day 1) or multiple doses (at Days 3, 7, 12, 18, 22, 30 and 35) of topical berberine-β-glycerophosphate cream administration is shown in Figure 4 (a and b, respectively). PK and DK parameters obtained using NCA after the first application (Day 1) and at Day 10 are summarized in Table 4.

**Table 4.** PK parameters in non-infected mice after IV administration of a berberine-β-glycerophosphate (BER-GP) solution (7.5 mg/kg) or in blood and skin of non-infected and *L. major*-infected BALB/c mice after a single topical treatment with a BER-GP cream at dose of 7.5 mg/kg (Day 1) or after chronic BER-GP cream administration twice daily during 9 days (Day 10)

Parameter	IV non-infected	Topical non-infected			Topical *L. major*		
	Day 1	Day 1		Day 10	Day 1		Day 10
	Plasma	Skin	Plasma	Plasma	Skin	Plasma	Plasma
*t*_½_ (h)	12.9 ± 3.5	7.5 ± 6.1	1.5 ± 0.4	3.5 ± 3.1	18.0 ± 5.3	9.7 ± 6.4	5.6 ± 2.9
*T*_max_ (h)	0.25	1	2	1	1	2	1
*C*_max_ (ng/mL)	99.2 ± 36.0	9.7 ± 4.9	17.8 ± 6.3	47.5 ± 29.3	25.3 ± 7.6	14.0 ± 3.1	89.2 ± 62.8
AUC*_t_* (ng/mL·h)	504.6 ± 213.8 (24 h)	27.4 ± 6.5 (12 h)	42.9 ± 16.7 (24 h)	262.8 ± 142.0 (12 h)	154.9 ± 19.4*** (12 h)	85.5 ± 43.6 (24 h)	448.2 ± 253.0 (12 h)
AUC_∞_ (ng/mL·h)	976.8 ± 326.7	28.1 ± 3.4	44.2 ± 16.8	328.4 ± 203.3	335.4 ± 189.9**	123.8 ± 66.9	608.7 ± 306.9
F (%)	100		4.5			12.7	

AUC*_t_*, area under the plasma concentration–time curve from time = 0 to time = *t*; AUC_∞_, area under the plasma concentration–time curve from time = 0 to time = infinity; F, bioavailability, calculated as: (AUC_topical_ × Dose_IV_)/(AUC_IV_ × Dose_topical_). Data were calculated using NCA and expressed as mean ± SD (*n* = 3–5). Comparisons between two groups were made by a parametric *t*-test comparing (i) skin of non-infected versus *L. major*-infected mice (** *P <* 0.01, *** *P <* 0.001); (ii) plasma of non-infected versus *L. major*-infected mice at Day 1 (no significant results) and (iii) plasma of non-infected versus *L. major*-infected mice at Day 10 (no significant results). ** *P <* 0.01, *** *P <* 0.001.

Either in healthy or infected mice, berberine concentration quantified in skin and plasma was higher at Day 10 (after several administrations) than at Day 1, probably due to the time required to achieve steady state more than drug accumulation, as *C*_trough_ remained constant from Day 3 to the end of the experiment (Figure 4). As expected and evidenced in Figure 2a, the infection seriously disrupted the barrier integrity of skin^11^ and 2-fold more berberine was quantified in the plasma of infected mice than in healthy ones (AUC of 448.2 versus 262.8 ng/mL·h, respectively, at Day 10; not significant; Table 4). The drug ratio between the skin and plasma^26^ was also changed by the infection, from around 1:2 in healthy versus 1:1 in damaged skin (comparison made once achieved the steady state; *P <* 0.001; Table 4). Berberine-β-glycerophosphate, with a logP value of −0.04 (experimental determination) would have more affinity with infected skin, a more hydrophilic substrate than intact skin, mostly due to stratum corneum removal and inflammation. Accordingly, the infection doubled the *t*_½_ of berberine in the skin (18.0 versus 7.5 h; Table 4).

After topical berberine administration (Day 1), the bioavailability was around 4% in healthy mice and 12% in infected ones (Table 4). However, these values diminished to around 1% or lower when expressed as percentage of applied dose. This issue highlights rapid liver clearance as the major disadvantage for berberine systemic bioavailability, which is a very helpful property if dermal, but not systemic, drug delivery is intended. The bioavailability of berberine has also been previously described to be much higher after its topical administration than after its oral administration in rats.^27^

The equilibrium of free drug fraction among different body compartments (in this case among plasma and skin, and then inside macrophages) is a common assumption in order to establish PK/PD relationships.^28^ Since 74.8% ± 5.0% of berberine binding to mice plasma proteins was calculated, we inferred that the *in vivo* efficacy of berberine with an unbound plasma concentration was 0.02 μM, lower than berberine *in vitro f*EC_50_ after 24 h (0.39 μM; Table S2) but between the *f*EC_50_ (0.01 μM) and *f*EC_90_ (0.08 μM) values estimated at longer incubation times (Table S2 and Figure 4a).

### Biochemical analysis after topical berberine-β-glycerophosphate administration: in vivo toxicity studies

Glucose, TRIG and cholesterol (CHO, HDL and LDL) levels, which are normally increased in several metabolic disorders and reduced after berberine administration,^29^ did not change at the end of berberine-β-glycerophosphate cream treatment (Table 5), neither in non-infected nor *L. major*-infected mice. Moreover, the levels obtained for the two transaminases (ALT and AST) and ALP were similar in control and treated mice, indicating correct liver function (Table S4). CRE and BUN levels, measuring kidney correct function, were also similar among groups (Table S4). All of these values are within the normal values reported from Charles Rivers for healthy BALB/c mice.^30^

**Table 5.** Biochemical parameters of non-infected and *L. major*-infected mice after treatment with berberine-β-glycerophosphate (BER-GP) cream compared with untreated control mice

Parameter	Non-infected		*L. major*	
	Control	BER-GP cream	Control	BER-GP cream
TRIG (mg/dL)	114.8 ± 7.4	256.8 ± 87.0	82.5 ± 14.8	100.7 ± 20.7
CHO (mg/dL)	104.4 ± 8.6	130.3 ± 13.6	90.0 ± 9.1	113.1 ± 8.4
HDL (mmol/L)	2.4 ± 0.1	2.3 ± 0.3	1.8 ± 0.1	2.3 ± 0.1
LDL (mmol/L)	0.4 ± 0.0	0.3 ± 0.0	0.3 ± 0.1	0.4 ± 0.1

Results are expressed as mean ± SD (*n* = 5). Multiple comparisons between groups were made by a non-parametric Kruskal–Wallis test followed by Dunn’s multiple comparisons test (no significant results).

## Discussion

This work analysed the efficacy of a topical berberine cream in a murine model of CL produced by *L. major*. At the end of treatment, the smaller lesion size of berberine-treated mice correlated with a decrease of 99.9% (4 log) in the parasite load (Figure 2d), lower inflammatory cell infiltration (especially neutrophils) (Figure 3a and b) and up-regulation of genes involved in the process of skin repair such as IL-10 or Arg-1 (Figure 3c).

In mice models, and more especially in humans,^31^ lesion evolution depends on parasite burden but it is also influenced by the inflammatory response.^32^ Therefore, the immune response could affect parasite survival and critically determine tissue damage and healing of lesions as well as response to treatment.^33^ In fact, in humans, immune mediators (such as TNF-α or granzyme B) and not the parasite are considered as the main factor responsible for tissue damage^34^ and there are many efforts addressing the unravelling of immunological pathways able to prevent tissue destruction without affecting parasite load.^35^ Drugs with dual leishmanicidal and anti-inflammatory effects would benefit both ‘clinical cure’ and ‘parasite clearance’, preventing tissue scarring, residual lesions and future reactivation of the infection. Berberine showed both properties, as both anti-*Leishmania* and anti-inflammatory effects were observed at the end of treatment. However, it cannot be elucidated whether the effect was produced directly or indirectly by the leishmanicidal effect of berberine (the leishmanicidal effect would occur before the healing process), as these determinations were only carried out at the endpoint and not during the course of the treatment.^36^ However, berberine is more widely known for its anti-inflammatory properties.^37^ In this context, the C57BL/6 mouse strain would be more suitable to evaluate the role of the anti-inflammatory potential of berberine in the course of CL lesions.^38^

PK/PD indices have been established as a very useful tool for optimizing the efficacy and administration schedule of common antimicrobials. However, PK/PD relationships are not well characterized for anti-leishmanial agents^39,40^ and far less after their topical application.^41,42^ The efficacy should be determined by the drug concentration inside dermal macrophages.^43^ An approach to determining this is through the determination of the free drug concentration in the dermis, which requires laborious techniques such as microdialysis.^28,44^ In the absence of this information, the free drug plasma concentration can be considered as the best surrogate correlation.^45^ There are previous studies confirming this matter in CL.^26,46^ However, the observed *in vivo* efficacy of berberine (around 90% parasite reduction in the skin; Figure 2d) was higher than what could be deduced from unbound plasma drug concentration (PK parameter) (*C*_trough_ at steady state around 0.02 μM) and *in vitro f*EC_50_ obtained even after 72 h incubation time (PD parameter) (0.01 μM; Table S2). In detail, from *in vitro* time-to-kill data, a drug concentration higher than 0.05 μM would be required to achieve 70% parasite clearance (Figure 1a).

This discrepancy between *in vitro* potency and *in vivo* efficacious concentrations could be due to multiple reasons.^47^ First, we did not measure the unbound drug, but total drug concentrations were corrected according to the extent of protein binding measured *in vitro*. Second, free plasma concentrations may represent a reliable surrogate for drug exposure inside macrophages, assuming rapid equilibrium between membranes and passive diffusion.^47^ Berberine has been described as a substrate for P-gp proteins.^48^ Moreover, berberine is a cationic molecule and its intracellular accumulation inside the lysosomes of macrophages could be higher than detected in plasma, as previously described for azithromycin.^49^ Third, the *in vivo* berberine activity could involve participation of the immune system, not represented in the *in vitro* studies.^36^ Finally, we can also speculate about the contribution of some berberine metabolites (with demonstrated *in vitro* antileishmanial activity, such as oxyberberine, 8-cyanodihydroberberine or tetrahydroberberine)^50^ to the effect observed *in vivo.* Berberine anti-diabetic effects were actually observed with plasma concentration of berberine after oral administration far below the concentration required to modulate insulin-signalling pathways *in vitro.*^13,51^

Berberine, as its sulphate salt, has been already tested in an *L. major*-BALB/c mice model.^52^ The treatment was not able to stop lesion progression after 10 days of treatment. The possible reasons for this disagreement would be: (i) we evaluated another salt of the drug; (ii) we have included the terpenoid menthol as permeation enhancer, and (iii) we treated the mice for a longer time. An additional antileishmanial effect of menthol cannot be excluded.

Overall, berberine-β-glycerophosphate salt in a cream containing 2.5% of menthol avoided parasite progression and favoured the lesion healing of *L. major*-infected BALB/c mice. The schedule of topical berberine application twice daily at a dose of 7.5 mg/kg did not show any ‘off-target’ effects (Table 5 and Table S4) such as lipid lowering or glucose regulation, as currently seen after oral administration of berberine. Pending tasks are to determine the suitable PK driver for berberine efficacy and find the best surrogate marker for it. On the other hand, the data on berberine systemic bioavailability (quite high) after topical administration of the cream (Table 4) allow us to reconsider the need to incorporate menthol as a permeation enhancer in the formulation.

## Supplementary Material

dkac007_Supplementary_Data

## Supplementary data

Supplementary methods, Tables S1 to S4 and Figure S1 are available as Supplementary data at *JAC* Online.

## References

[1] Bailey F , Mondragon-Shem K, Hotez P et al A new perspective on cutaneous leishmaniasis—implications for global prevalence and burden of disease estimates. PLoS Negl Trop Dis 2017; 11: e0005739. 28796782 10.1371/journal.pntd.0005739 PMC5552022

[2] Azim M , Khan SA, Ullah S et al Therapeutic advances in the topical treatment of cutaneous leishmaniasis: a review. PLoS Negl Trop Dis 2021; 15: e0009099. 33657097 10.1371/journal.pntd.0009099 PMC7928440

[3] Burza S , Croft SL, Boelaert M. Leishmaniasis – Authors’ reply. Lancet 2019; 393: 872–3. 10.1016/S0140-6736(18)33057-5 30837140

[4] DNDi . Target product profile for cutaneous leishmaniasis. https://dndi.org/diseases/cutaneous-leishmaniasis/target-product-profile/.

[5] Singh IP , Mahajan S. Berberine and its derivatives: a patent review (2009–2012). Expert Opin Ther Pat 2013; 23: 215–31. 23231038 10.1517/13543776.2013.746314

[6] Imenshahidi M , Hosseinzadeh H. *Berberis vulgaris* and berberine: an update review. Phytother Res 2016; 30: 1745–64. 27528198 10.1002/ptr.5693

[7] Och A , Podgórski R, Nowak R. Biological activity of berberine—a summary update. Toxins 2020; 12: 713. 10.3390/toxins12110713 PMC7697704 33198257

[8] Saha P , Bhattacharjee S, Sarkar A et al Berberine chloride mediates its anti-leishmanial activity via differential regulation of the mitogen activated protein kinase pathway in macrophages. PLoS One 2011; 6: e18467. 21483684 10.1371/journal.pone.0018467 PMC3071726

[9] Calvo A , Moreno E, Larrea E et al Berberine-loaded liposomes for the treatment of *Leishmania infantum*-infected BALB/c mice. Pharmaceutics 2020; 12: 858. 10.3390/pharmaceutics12090858 PMC7558179 32916948

[10] Spinozzi S , Colliva C, Camborata C et al Berberine and its metabolites: relationship between physicochemical properties and plasma levels after administration to human subjects. J Nat Prod 2014; 77: 766–72. 24593257 10.1021/np400607k

[11] Van Bocxlaer K , Yardley V, Murdan S et al Drug permeation and barrier damage in *Leishmania*-infected mouse skin. J Antimicrob Chemother 2016; 71: 1578–85. 26903275 10.1093/jac/dkw012

[12] Chiang A , Tudela E, Maibach HI. Percutaneous absorption in diseased skin: an overview. J Appl Toxicol 2012; 32: 537–63. 22912973 10.1002/jat.1773

[13] Wang K , Feng X, Chai L et al The metabolism of berberine and its contribution to the pharmacological effects. Drug Metab Rev 2017; 49: 139–57. 28290706 10.1080/03602532.2017.1306544

[14] Tey HL , Tay EY, Tan WD. Safety and antipruritic efficacy of a menthol-containing moisturizing cream. Skinmed 2017; 15: 437–9. 29282180

[15] Silva A , Scher R, Santos FV et al Leishmanicidal activity and structure-activity relationships of essential oil constituents. Molecules 2017; 22: 815. 10.3390/molecules22050815 PMC6154737 28509873

[16] Patel RK , Patel NA, Patel NJ et al The formulation and evaluation of topical berberine-hydrochloride products. Pharm Tech 2010; 34: 60–9.

[17] Gattu S , Maibach HI. Enhanced absorption through damaged skin: an overview of the *in vitro* human model. Skin Pharmacol Physiol 2010; 23: 171–6. 20185974 10.1159/000288163

[18] Hendrickx S , Eberhardt E, Mondelaers A et al Lack of correlation between the promastigote back-transformation assay and miltefosine treatment outcome. J Antimicrob Chemother 2015; 70: 3023–6. 26253089 10.1093/jac/dkv237

[19] Odds FC . Synergy, antagonism, and what the chequerboard puts between them. J Antimicrob Chemother 2003; 52: 1. 12805255 10.1093/jac/dkg301

[20] Dow N . Determination of compound binding to plasma proteins. Curr Protoc Pharmacol 2006; Chapter 7: Unit 7.5. 10.1002/0471141755.ph0705s34 22294178

[21] OECD . Test No. 428: Skin absorption: *in vitro* method, 2004. https://www.oecd-ilibrary.org/environment/test-no-428-skin-absorption-in-vitro-method_9789264071087-en.

[22] Zhang Y , Huo M, Zhou J et al PKSolver: an add-in program for pharmacokinetic and pharmacodynamic data analysis in Microsoft Excel. Comput Methods Programs Biomed 2010; 99: 306–14. 20176408 10.1016/j.cmpb.2010.01.007

[23] Gonzalez D , Schmidt S, Derendorf H. Importance of relating efficacy measures to unbound drug concentrations for anti-infective agents. Clin Microbiol Rev 2013; 26: 274–88. 23554417 10.1128/CMR.00092-12 PMC3623378

[24] Chen J , Jiang Q-D, Chai Y-P et al Natural terpenes as penetration enhancers for transdermal drug delivery. Molecules 2016; 21: 1709. 10.3390/molecules21121709 PMC6273457 27973428

[25] Vanti G , Wang M, Bergonzi MC et al Hydroxypropyl methylcellulose hydrogel of berberine chloride-loaded escinosomes: dermal absorption and biocompatibility. Int J Biol Macromol 2020; 164: 232–41. 32682035 10.1016/j.ijbiomac.2020.07.129

[26] Wijnant G-J , Croft SL, de la Flor R et al Pharmacokinetics and pharmacodynamics of the nitroimidazole DNDI-0690 in mouse models of cutaneous leishmaniasis. Antimicrobial Agents Chemother 2019; 63: e00829-19. 10.1128/AAC.00829-19 PMC6709472 31262757

[27] Buchanan B , Meng Q, Poulin M-M et al Comparative pharmacokinetics and safety assessment of transdermal berberine and dihydroberberine. PLoS One 2018; 13: e0194979. 29579096 10.1371/journal.pone.0194979 PMC5868852

[28] Muller M , dela Pena A, Derendorf H. Issues in pharmacokinetics and pharmacodynamics of anti-infective agents: distribution in tissue. Antimicrob Agents Chemother 2004; 48: 1441–53. 15105091 10.1128/AAC.48.5.1441-1453.2004 PMC400530

[29] Xu X , Yi H, Wu J et al Therapeutic effect of berberine on metabolic diseases: both pharmacological data and clinical evidence. Biomed Pharmacother 2021; 133: 110984. 33186794 10.1016/j.biopha.2020.110984

[30] Charles River . BALB/C mouse biochemistry. https://www.criver.com/sites/default/files/resources/BALBcMouseClinicalPathologyData.pdf.

[31] Saldanha MG , Pagliari C, Queiroz A et al Tissue damage in human cutaneous leishmaniasis: correlations between inflammatory cells and molecule expression. Front Cell Infect Microbiol 2020; 10: 355. 32766167 10.3389/fcimb.2020.00355 PMC7381142

[32] Scott P , Novais FO. Cutaneous leishmaniasis: immune responses in protection and pathogenesis. Nat Rev Immunol 2016; 16: 581–92. 27424773 10.1038/nri.2016.72

[33] Navas A , Fernandez O, Gallego-Marin C et al Profiles of local and systemic inflammation in the outcome of treatment of human cutaneous leishmaniasis caused by *Leishmania* (*Viannia*). Infect Immun 2020; 88: e00764–19. 31818959 10.1128/IAI.00764-19 PMC7035935

[34] Nylén S , Eidsmo L. Tissue damage and immunity in cutaneous leishmaniasis. Parasite Immunol 2012; 34: 551–61. 23009296 10.1111/pim.12007

[35] Novais FO , Nguyen BT, Scott P. Granzyme B inhibition by tofacitinib blocks the pathology induced by CD8 T cells in cutaneous leishmaniasis. J Invest Dermatol 2021; 141: 575–85. 32738245 10.1016/j.jid.2020.07.011 PMC7855313

[36] Gómez MA , Navas A, Prieto MD et al Immuno-pharmacokinetics of meglumine antimoniate in patients with cutaneous leishmaniasis caused by *Leishmania* (*Viannia*). Clin Infect Dis 2021; 72: e484–92. 32818964 10.1093/cid/ciaa1206 PMC8130027

[37] Song D , Hao J, Fan D. Biological properties and clinical applications of berberine. Front Med 2020; 14: 564–82. 32335802 10.1007/s11684-019-0724-6

[38] Mears ER , Modabber F, Don R et al A review: the current *in vivo* models for the discovery and utility of new anti-leishmanial drugs targeting cutaneous leishmaniasis. PLoS Negl Trop Dis 2015; 9: e0003889. 26334763 10.1371/journal.pntd.0003889 PMC4559374

[39] Voak AA , Harris A, Coteron-Lopez JM et al Pharmacokinetic/pharmacodynamic relationships of liposomal amphotericin B and miltefosine in experimental visceral leishmaniasis. PLoS Negl Trop Dis 2021; 15: e0009013. 33651812 10.1371/journal.pntd.0009013 PMC7924795

[40] Kip AE , Castro MDM, Gomez MA et al Simultaneous population pharmacokinetic modelling of plasma and intracellular PBMC miltefosine concentrations in New World cutaneous leishmaniasis and exploration of exposure–response relationships. J Antimicrob Chemother 2018; 73: 2104–11. 29757380 10.1093/jac/dky143 PMC6251527

[41] Van Bocxlaer K , Gaukel E, Hauser D et al Topical treatment for cutaneous leishmaniasis: dermato-pharmacokinetic lead optimization of benzoxaboroles. Antimicrobial Agents Chemother 2018; 62: e02419-17. 10.1128/AAC.02419-17 PMC5923108 29507073

[42] Caridha D , Vesely B, van Bocxlaer K et al Route map for the discovery and pre-clinical development of new drugs and treatments for cutaneous leishmaniasis. Int J Parasitol Drugs Drug Resist 2019; 11: 106–17. 31320296 10.1016/j.ijpddr.2019.06.003 PMC6904839

[43] Voak AA , Standing JF, Sepulveda N et al Pharmacodynamics and cellular accumulation of amphotericin B and miltefosine in *Leishmania donovani*-infected primary macrophages. J Antimicrob Chemother 2018; 73: 1314–23. 29506127 10.1093/jac/dky014 PMC5909632

[44] Carryn S , Chanteux H, Seral C et al Intracellular pharmacodynamics of antibiotics. Infect Dis Clin North Am 2003; 17: 615–34. 14711080 10.1016/s0891-5520(03)00066-7

[45] Mouton JW , Theuretzbacher U, Craig WA et al Tissue concentrations: do we ever learn? J Antimicrob Chemother 2008; 61: 235–7. 18065413 10.1093/jac/dkm476

[46] Voelkner NMF , Voelkner A, Costa J et al Dermal pharmacokinetics of pyrazinamide determined by microdialysis sampling in rats. Int J Antimicrob Agents 2018; 51: 190–6. 29032112 10.1016/j.ijantimicag.2017.10.001

[47] Smith DA , Di L, Kerns EH. The effect of plasma protein binding on *in vivo* efficacy: misconceptions in drug discovery. Nat Rev Drug Discov 2010; 9: 929–39. 21119731 10.1038/nrd3287

[48] Zhang Y , Guo L, Huang J et al Inhibitory effect of berberine on broiler P-glycoprotein expression and function: *in situ* and *in vitro* studies. Int J Mol Sci 2019; 20: 1966. 10.3390/ijms20081966 PMC6515058 31013627

[49] Matzneller P , Krasniqi S, Kinzig M et al Blood, tissue, and intracellular concentrations of azithromycin during and after end of therapy. Antimicrob Agents Chemother 2013; 57: 1736–42. 23357769 10.1128/AAC.02011-12 PMC3623349

[50] Vennerstrom JL , Lovelace JK, Waits VB et al Berberine derivatives as antileishmanial drugs. Antimicrobial Agents Chemother 1990; 34: 918–21. 10.1128/aac.34.5.918 PMC171721 2360830

[51] Zhang JW , Zhou F, Lu M et al Pharmacokinetics-pharmacology disconnection of herbal medicines and its potential solutions with cellular pharmacokinetic-pharmacodynamic strategy. Curr Drug Metab 2012; 13: 558–76. 22475335 10.2174/1389200211209050558

[52] El-On J , Jacobs GP, Witztum E et al Development of topical treatment for cutaneous leishmaniasis caused by *Leishmania major* in experimental animals. Antimicrobial Agents Chemother 1984; 26: 745–51. 10.1128/aac.26.5.745 PMC180006 6517557

