# Supplementary material for: Effect of topical berberine in murine cutaneous leishmaniasis lesions
Source: J Antimicrob Chemother. 2022 Jan 28;77(4):1072–81. doi: 10.1093/jac/dkac007 (PMC9000957; doi:10.1093/jac/dkac007)
Supplement: dkac007_Supplementary_Data [file dkac007_supplementary_data.docx]

**Supplementary data**

**Material and methods**

***Chemicals***

Berberine chloride, dimethylsulfoxide (DMSO), 3-[4,5-dimethylthiazol-2-yl]-2,5-diphenyltetrazolium bromide (MTT), β-glycerophosphate disodium salt hydrate, 4-(2-hydroxyethyl)piperazine-1-ethanesulfonic acid sodium salt (HEPES), adenine, haemin, biotin, menthol, Polysorbate^®^ 20, verapamil hydrochloride, Nipajin^TM^, ethylenediamine tetraacetic acid (EDTA) and n-octanol were obtained from Sigma-Aldrich (St. Louis, MO, Canada). Stearic acid, cetyl alcohol, glycerol monoestearate and solid paraffin were purchased by Fagron (Terrassa, Spain). Liquid paraffin was obtained from Guinama (La Pobla de Valbona, Spain). All other reagents were of analytical grade.

***Parasites and cells***

*Leishmania major* promastigotes (clone VI, MHOM/IL/80/Friendlin) were maintained at 26 °C in continuing stirred M199 1X medium (Sigma) supplemented with 10% heat-inactivated fetal bovine serum (FBS) (Gibco, Gaithersburg, MD, USA), 25 mM HEPES (pH 7.2), 0.1 mM adenine, 0.0005% (w/v) haemin, 0.0001% (w/v) biotin, and 100 UI/mL penicillin and 100 mg/mL streptomycin (Gibco) or in Schneider´s modified medium (Sigma) supplemented with 10% FBS and 100 UI/mL penicillin and 100 mg/mL streptomycin. Metacyclic promastigotes were obtained from stationary cultures by treatment with 20 µg/mL of peanut agglutinin (PNA, 5 mg/mL in PBS) and were used for infections. Bone marrow derived macrophages (BMDM) were isolated from BALB/c mice by flushing the femur and tibia with PBS. For differentiation, cells were then placed in DMEM medium (Gibco) supplemented with 10% FBS, 1% penicillin/streptomycin and 20% filtered supernatant from L929 cell line, as source of granulocyte-macrophage colony stimulating factor (GM-CSF), and incubated for 8 days at 37 °C and 5% CO_2_ with medium change every 3 days.

***Animals***

BALB/c mice (Harlan, Spain) weighing approximately 20 g were kept under conventional conditions with free access to food and water. Mice were infected by subcutaneous (s.c.) inoculation of 10^5^ infective metacyclic promastigotes of *L. major* in the base of the tail. Two weeks after infection, mice were randomly sorted into different groups, each group having a mean lesion size of 6 mm^2^ and treatments were started with vehicle or berberine-β-glycerophosphate cream.

***Preparation of berberine-β-glycerophosphate salt***

Firstly, a phase solubility study was carried out according to the method previously reported by Higuchi[^1^](#_ENREF_1) using an excess of berberine in aqueous solutions containing increasing amounts of β-glycerophosphate (0-120 mM). As a linear type system was deducted by the shape and slope of the solubility diagram, an interaction in a molar ratio 1:1 (berberine-β:glycerophosphate) was determined. Thus, 10 mg of berberine were mixed with 6 mg of β-glycerophosphate in 7.5 mL of distilled water and allowed to dissolve for 5 min at room temperature. Finally, the resulting salt was lyophilized and stored at 4 °C protected from light. Log*P* values of berberine and berberine-β-glycerophosphate salt were determined experimentally following the shake flask method according to the OECD guidelines.[^2^](#_ENREF_2) Briefly, saturated solutions of berberine and berberine-β-glycerophosphate salt were placed in n-octanol and water. Mixtures were left under continuous stirring for 48 h at 25 °C for equilibrium. Then, mixtures were centrifuged at 5250 *g* for 15 min and filtered using 0.45 µm-membrane filters. Finally, the concentration of solubilized berberine in each solvent was quantified by UV-visible at λ_max_ 346 nm. This berberine-β-glycerophosphate salt showed an aqueous solubility of 5.01 mM and a log*P* of - 0.04, while berberine presented values of 4.58 mM and 0.14, respectively.

***Back transformation assay (BTA)***

The activity of berberine-β-glycerophosphate and menthol (alone or in combination) against *L. major* amastigotes was evaluated by the BTA at 48 h, as previously reported.[^3^](#_ENREF_3) Briefly, 5 × 10^4^ BMDM were seeded in 96-well plates and incubated at 37 °C for 24 h in DMEM medium. Then, cells were infected with stationary promastigotes of *L. major* in a proportion 10:1 (parasite:macrophage) and incubated with parasites overnight at 37 °C and 5% CO_2_. After this, cells were washed at least three times with pre-heated complete DMEM and treated with different concentrations of drugs. After incubation times, medium was removed, and plates were incubated at 26 °C with complete Schneider’s modified medium to favor the release of remaining viable amastigotes. After 7 days, wells containing medium with remaining parasites were transferred to new 96-well plates and the MTT assay was performed.

***Drug combination studies***

The fractional inhibitory concentration index (FICI) was used to describe the interaction between berberine-β-glycerophosphate and menthol *in vitro*, after 48 h. For that, starting from the EC_50_ of each compound, two higher concentrations (EC_50_ x 2, EC_50_ x 4) and two lower concentrations (EC_50_ / 2 and EC_50_ / 4) were set. For each combination, FICI was calculated according to the formula: FIC_A_ = EC_50_ compound A in combination with compound B/ EC_50_ compound A alone; FIC_B_ = EC_50_ compound B in combination with compound A/ EC_50_ compound B alone. Then, the sum of all FICs (SFIC) for each combination was calculated (SFIC = FIC compound A + FIC compound B). Finally, FICI value was calculated (SFIC divided by de number of the different combinations). FICI was used to describe the compound interaction effects *in vitro*. Synergy was defined as FICI < 0.5, additivity as 0.5 < FICI < 4, and antagonism as FICI > 4.[^4^](#_ENREF_4)

***Characterization and stability of berberine-β-glycerophosphate cream***

For the characterization of the vehicle and berberine-β-glycerophosphate cream, viscosity measurements were carried out at 32 °C using a Haake Viscotester 550 rotational viscometer with a SV2 rotor (Karlsruhe, Germany) and equipped with a thermostatic bath Thermo Phoenix II (Karlsruhe, Germany). Measures were taken from 0 to 700 1/s. Viscosities at 150 1/s were chosen for comparison and are expressed as Pascal per second (Pa·s). pH was measured using a digital pH meter (GPL 21, Crison Instruments, Barcelona, Spain). Briefly, 500 mg of the creams were uniformly dispersed in 50 mL of distilled water, and afterwards, pH was determined. Spreadability test was carried out using the parallel plate method with modifications.[^5^](#_ENREF_5) First, 100 mg of the creams were placed within a circle of 1 cm diameter premarked on a glass plate, over which a second glass plate was placed. A 10 g weight was allowed to rest on the upper glass plate for 5 min. The increase in the diameter due to spreading was noted. All determinations were carried out in triplicate. Organoleptic characteristics, in terms of odour, colour, texture, consistency and external appearance were also recorded. Finally, the stability assessment of berberine-β-glycerophosphate cream was carried out over an 8-months period of storage at three different temperatures: 4, 25, and 40 °C. On months 3 and 8, pH, spreadability, organoleptic properties, phase separation, drug precipitation and gravitational stability were evaluated and compared with the initial parameters. For the determination of berberine content, 50 mg of the formulations were mixed with methanol overnight at 32 °C. Then, samples were centrifuged at 5250 *g* for 15 min and berberine was quantified by UV-visible. To check the accuracy of the method, blank creams with different amounts of berberine-β-glycerophosphate were also analysed and the recovery percentage was found to be 96-102%. For the gravitational stability test, berberine-β-glycerophosphate cream was centrifuged at 25 °C and 2700 *g* for 30 min to assess accelerated deterioration.[^6^](#_ENREF_6)

***Ex vivo permeation studies in healthy mouse skin***

Franz diffusion cells devices (MicroettePlusTM, Hanson Research Crop., Chatsworth, CA, USA) were used to determine the permeation of berberine-β-glycerophosphate through freshly excised mice skin, according to OECD guideline 428.[^7^](#_ENREF_7) Two different creams were evaluated: i) berberine-β-glycerophosphate (0.5%) and ii) berberine-β-glycerophosphate (0.5%) + menthol (2.5%). Transcutaneous diffusion was assessed according to OECD guideline.[^7^](#_ENREF_7) Receptor compartments were filled with a PBS solution (4.5 mL) and were maintained with continuous stirring (400 rpm) at 32 ± 1 °C. Full-thickness excised female mouse skin pieces were inspected for macroscopic damage and fat tissue was carefully removed using forceps. Then, skin pieces were placed between the donor and receptor compartments with the stratum corneum side up and kept in place with a clamp. Experiments were conducted under infinite dose conditions: 500 mg of the berberine-β-glycerophosphate creams were placed on 0.64 cm^2^ of skin in the donor compartment. Receptor fluid samples were taken by manual sampling of 1 mL aliquots at determined times (0.5, 1, 2, 4, 6, 8, 10 and 24 h). Each sampled aliquot was replaced with the same fresh volume of receptor phase. Permeation parameters were estimated: The flux of drug permeated was calculated from the slope of the steady-state portion of the permeation profile by linear regression analysis. The lag time (h) was calculated from the back extrapolation of the steady-state portion of the graph. The permeability coefficient was also calculated as *Jss*/Cdonor, with Cdonor being the drug concentration applied to the skin surface.

When the experiment was finished, berberine in the receptor compartment was quantified using high performance liquid chromatography tandem mass spectrometry (HPLC-MS/MS), after diluting with acetonitrile.

***DNA extraction and parasite quantification***

The Macherey-Nagel NucleoSpin® Tissue kit was used as per manufacturer's instructions to isolate DNA from skin lesion and LN of infected mice. The parasite burden was measured by qRT-PCR of 10 ng of total DNA (quantified by NanoDrop (NanoDrop Technologies ND-1000 UV-Vis Spectrophotometer), using the iQ^TM^ SYBR® Green Supermix (Bio-Rad) and specific primers for minicircle kinetoplastic DNA (kDNA) of *Leishmania* (Leish kDNA, Table S1) by the CFX96 real time PCR detection system (Bio-Rad, Hercules, CA, USA). The number of kDNA copies was determined by extrapolation from the cycle threshold of each sample on a standard curve of known concentrations. The standard was generated by insertion of the *Leishmania* amplicon in a pCR2.1-TOPO vector (TOPO TA cloning kit; Invitrogen, Carlsbad, CA, USA). Results are expressed as number of copies of the plasmid/10 ng of total DNA.

***RNA extraction and cytokine expression***

Cytokines expression in skin lesions of infected mice was determined by qRT-PCR. RNA was extracted following the TRI Reagent® protocol. Then, 1µg of RNA was treated with DNase (Gibco-BRL) prior to reverse transcription with M-MLV reverse transcriptase (GibcoBRL) in the presence of RNase OUT (Gibco-BRL). Q-RT PCR was performed with iQ SYBR Green supermix (Bio-Rad) in a CFX96 system from Bio-Rad, using specific primers for each gene (see Table S1). To determine specificity, final PCR products were analysed by melting curves and electrophoresis. Results were normalised to β-actin. The amount of each transcript was expressed by the formula: 2^ct(β-actin)-ct(gene)^, with ct being the point at which the fluorescence rises appreciably above the background fluorescence.

**Table S1.** Primers used in this study.

|  | **Sense primer (5´-3´)** | **Antisense primer (5´-3´)** |
| --- | --- | --- |
| **β-actin** | CGCGTCCACCCGCGAG | CCTGGTGCCTAGGGCG |
| **Leish 18S** | CCAAAGTGTGGAGATCGAAG | GGCCGGTAAAGGCCGAATAG |
| **Leish kDNA** | CCTATTTTACACCAACCCCCAGT | GGGTAGGGGCGTTCTGCGAAA |
| **TNF-α** | CTTCCAGAACTCCAGGCGGT | GGTTTGCTACGACGTGGG |
| **COX-2** | CAAAAGCTGGGAAGCCTTCTC | CCTCGCTTATGATCTGTCTTG |
| **IL-1β** | GCCACCTTTTGACAGTGATG | TAATGGGAACGTCACACACC |
| **CCL3** | TGCAACCAAGTCTTCTCAGC | TCAGTTCCAGGTCAGTGATG |
| **CCL2** | CAGAATTGCTTGAGGTGGTTGTG | CAGAAGTGCTTGAGGTGGTTGTG |
| **CCL17** | TGAGGTCACTTCAGATGCTG | GGACAGTCAGAAACACGATG |
| **CCL22** | TCATTAGACACCTGACGAGG | CTTCCACATTGGCACCATAG |
| **CXCL1** | ACCTCAAGAACATCCAGAGC | ACTTGGGGACACCTTTTAGC |
| **XCL10** | AGTGCTGCCGTCATTTTCTG | GGATTCAGACATCTCTGCTC |
| **CCR5** | TACAAGAGACTCTGGCTC | GAATACCAGGGAGTAGAG |
| **Arg-1** | TGGGGAAAGCCAATGAAGAG | AGGAGAAAGGACACAGGTTG |
| **IL-10** | GGACAACATACTGCTAACCG | AATCACTCTTCACCTGCTCC |
| **TFG-β** | CGGCAGCTGTACATTGAC | TCAGCTGCACTTGCAGGAGC |

***Immunohistochemistry studies***

Immunohistochemistry was applied using the following primary antibodies once fixed sections were cut: rat anti-mouse F4/80 (1:400; eBiosciences, 14–4801), rat anti-mouse NIMP-R14 (1:10,000; Abcam, ab2557) and rabbit anti-CD3 (1:300; Thermo Scientific, RM9106). Antigen retrieval was performed treating the samples with 2 mg/mL proteinase K at 37 °C for 30 min (for F4/80, and NIMP-R14) or heating for 30 min at 95 °C in 0,01 M Tris-1 mM EDTA pH 9 in a Pascal pressure chamber (Dako, S2800) (for CD3). In the case of rat primary antibodies, sections were first incubated with rabbit anti-rat (Dako, E0468) secondary antibody. Then, the EnVisionTM system (Dako, K4011) was used in all cases according to manufacturer instructions. For each assay, digital images were scanned using a digital microscope system (Aperio ScanScope CS2, Leica Biosystems, Nussloch, Germany) and snapshots of higher magnification images were captured using image software (Aperio ImageScope, Leica Biosystems). Then, the percentage of area stained in each image was quantified by counting the number of pixels staining above a threshold intensity and normalising to the total number of pixels. The software Fiji 2.0. was used.

***In vitro determination of berberine binding to plasma and culture medium proteins***

To quantify berberine binding to proteins, different concentrations of berberine-β-glycerophosphate diluted in DMEM (from 25 to 100 ng/mL) or mouse plasma (from 50 to 1000 ng/mL) were incubated at 37 ºC for 20 min, and passed through 30 kDa MWCO amicones by centrifugation at 1000 *g* for 30 min. Berberine in the ultrafiltrate was quantified using high performance liquid chromatography tandem mass spectrometry (HPLC-MS/MS). Finally, the berberine-protein binding in the medium was calculated according to the following equation:

Plasma protein binding (%) = 100 - $\left[ \frac{\text{concentration in ultrafiltrate}}{\text{concentration added}}\text{ x 100} \right]$

***Berberine extraction from plasma and skin samples***

For quantifying plasma samples, 50 µL of plasma were treated with 400 µL of acetonitrile containing 1% (v/v) formic acid. Then, samples were gently vortexed and centrifuged at 6,000 *g* for 10 min. 350 µL of each sample were collected, put into vials, and assayed using HPLC-MS/MS. Calibration curves were also made in plasma in the range: 2.5-500 ng/mL. All curves met the previously established performance criteria (R ^2^ > 0.999, slopes significantly different from 0 and a relative error (in %) for calibrators < 15%). Verapamil hydrochloride was used as internal standard.

For berberine quantification in skin samples, skin was cut into small pieces, soaked in methanol and placed at 32 °C for 24 h under stirring to ensure effective extraction of the drug and sonicated for 1 h, as previously reported.[^8^](#_ENREF_8) Samples were then centrifuged at 8000 *g* for 20 min and supernatants were analysed for berberine content by HPLC-MS/MS. To check the accuracy of the method, skins with different amounts of berberine were also analysed and the recovery percentage was found to be 90-95%.

***Berberine quantification by HPLC-MS/MS***

After extraction procedures, HPLC-MS/MS was used to quantify berberine-protein binding in culture medium and plasma, berberine in the receptor compartment and in the skin after *ex vivo* permeation and penetration studies and the amount of berberine in plasma and skin after pharmacokinetic and dermatokinetic studies. HPLC-MS/MS analyses were operated using an Agilent 1200 liquid chromatography system equipped with an Agilent 6410 Triple Quadrupole MS system (Agilent Technologies, USA). The separation was performed on a XSelect® CSH^TM^ Phenyl-Hexyl column (150 mm x 4.6 mm, 3.5 μm) operating at 40 °C with a mobile phase consisting of a mixture of 0.1% (v/v) formic acid in acetonitrile and 0.1% (v/v) formic acid in type I water (proportion 30:70, respectively) using isocratic conditions, with a flux of 0.7 mL/min and a sample injection volume of 10 μL.

**Results**

**Table S2.** *In vitro* activity of berberine-β-glycerophosphate (BER-GP) against *L. major* amastigotes after 24, 48 and 72 h of treatment evaluated by the back transformation assay (BTA). Data are expressed as EC_50_, *f*EC_50_, EC_90_ and *f*EC_90_ in μM concentration (95% confidence interval, n = 4).

|  |  | *L. major* amastigotes | | |
| --- | --- | --- | --- | --- |
|  |  | 24 h | 48 h | 72 h |
| BER-GP | EC_50_ (µM) | 0.56 (0.36-0.87) | 0.07 (0.06-0.10) | 0.02 (0.02-0.03) |
|  | *f*EC_50_ (µM) | 0.39 (0.25-0.60) | 0.05 (0.04-0.07) | 0.01 (0.01-0.02) |
|  | EC_90_ (µM) | 1.52 (0.99-2.39) | 0.22 (0.16-0.30) | 0.12 (0.08-0.18) |
|  | *f*EC_90_ (µM) | 1.05 (0.69-1.66) | 0.15 (0.11-0.21) | 0.08 (0.06-0.12) |

Abbreviations: EC_50_ and EC_90_: 50 and 90% effective concentrations; *f*EC_50_ and *f*EC_90_: EC_50_ and EC_90_ corrected for protein binding.

**Figure S1.** Sigmoidal fitting of dose-response curves of berberine-β-glycerophosphate (BER-GP) salt alone or in combination with menthol (MNT), either at 10 or 50 µM, against *L. major* amastigotes after 48 h treatment evaluated by the BTA assay. Data expressed as mean ± SD (n = 3).

**Table S3.** Physicochemical characteristics of vehicle and berberine-β-glycerophosphate (BER-GP) cream after preparation. Data expressed as mean ± SD (n = 3).

| **Formulation** | Viscosity (Pa·s) | Spreadability (cm) | pH |
| --- | --- | --- | --- |
| **Vehicle** | 0.97 ± 0.15 | 1.28 ± 0.09 | 6.09 ± 0.29 |
| **BER-GP cream** | 1.29 ± 0.05 | 0.52 ± 0.03 | 4.81 ± 0.12 |

**Table S4**. Biochemical parameters of non-infected and *L. major*-infected mice after treatment with berberine-β-glycerophosphate (BER-GP) cream compared to untreated control mice. Results are expressed as mean ± SD (n = 5). Multiple comparisons between groups were made by a non-parametric Kruskal Wallis test followed by Dunn’s multiple comparisons test.

|  | non-infected | |  | *L. major* | |
| --- | --- | --- | --- | --- | --- |
|  | Control | BER-GP Cream |  | Control | BER-GP Cream |
| ALT (U/L) | 23.5 ± 2.3 | 37.7 ± 10.6 |  | 48.7 ± 25.2 | 38.9 ± 7.9 |
| AST (U/L) | 79.8 ± 25.0 | 108.1 ± 22.5 |  | 84.1 ± 18.4 | 89.0 ± 12.1 |
| ALP (U/L) | 133.3 ± 15.1 | 185.0 ± 46.1 |  | 84.6 ± 7.8 | 81.9 ± 5.5 |
| CRE (mg/dL) | 0.2 ± 0.0 | 0.3 ± 0.1 |  | 0.1 ± 0.0 | 0.2 ± 0.0 |
| GLUCOSE (mg/dL) | 158.7 ± 10.5 | 150.5 ± 32.1 |  | 172.0 ± 24.0 | 202.0 ± 30.9 |
| BUN (mg/dL) | 35.4 ± 2.1 | 34.5 ± 5.8 |  | 45.1 ± 2.4 | 42.6 ± 5.9 |

Abbreviations: ALT: alanine aminotransferase; AST: aspartate aminotransferase; ALP: alkaline phosphatase; CRE: creatinine; BUN: urea.

**References**

1. Higuchi T. A phase solubility technique. *Adv Anal Chem Instrum* 1965; **4**: 117-211.

2. OECD. *Test No. 107: Partition Coefficient (n-octanol/water): Shake Flask Method*, 1995.

3. Hendrickx S, Eberhardt E, Mondelaers A *et al.* Lack of correlation between the promastigote back-transformation assay and miltefosine treatment outcome. *J Antimicrob Chemother* 2015; **70**: 3023-6.

4. Odds FC. Synergy, antagonism, and what the chequerboard puts between them. *J Antimicrob Chemother* 2003; **52**: 1.

5. Cirri M, Bragagni M, Mennini N *et al.* Development of a new delivery system consisting in "drug--in cyclodextrin--in nanostructured lipid carriers" for ketoprofen topical delivery. *Eur J Pharm Biopharm* 2012; **80**: 46-53.

6. Anchisi C, Maccioni AM, Sinico C *et al.* Stability studies of new cosmetic formulations with vegetable extracts as functional agents. *Farmaco* 2001; **56**: 427-31.

7. OECD. *Test No. 428: Skin Absorption: In Vitro Method*, 2004.

8. Freag MS, Torky AS, Nasra MM *et al.* Liquid crystalline nanoreservoir releasing a highly skin-penetrating berberine oleate complex for psoriasis management. *Nanomedicine* 2019; **14**: 931-54.
